# A longitudinal follow-up of continuous laryngoscopy during exercise test scores in athletes irrespective of laryngeal obstruction, respiratory symptoms, and intervention

**DOI:** 10.1186/s13102-023-00681-9

**Published:** 2023-07-15

**Authors:** Tommie Irewall, Catharina Bäcklund, Estelle Naumburg, Marie Ryding, Nikolai Stenfors

**Affiliations:** 1 Division of Medicine, Department of Public Health and Clinical Medicine, Umeå University, Umeå, Sweden; 2 Unit of Physiotherapy, Östersund Hospital, Region Jämtland Härjedalen, Östersund, Sweden; 3 Department of Clinical Sciences, Pediatrics, Umeå University, Umeå, Sweden; 4 Unit of Otorhinolaryngology, Östersund Hospital, Region Jämtland Härjedalen, Östersund, Sweden

**Keywords:** EILO, Exercise-induced laryngeal obstruction, Vocal cord dysfunction, CLE test

## Abstract

**Background:**

Exercise-induced laryngeal obstruction (EILO) is diagnosed by the continuous laryngoscopy during exercise (CLE) test. Whether or how much CLE test scores vary over time is unknown. This study aimed to compare CLE test scores in athletes over time, irrespective of respiratory symptoms and grade of laryngeal obstruction.

**Methods:**

Ninety-eight athletes previously screened for EILO were invited for a follow-up CLE test irrespective of CLE scores and respiratory symptoms. Twenty-nine athletes aged 16–27 did a follow-up CLE test 3–23 months after the baseline test. Laryngeal obstruction at the glottic and supraglottic levels was graded by the observer during exercise, at baseline and follow-up, using a visual grade score (0–3 points).

**Results:**

At baseline, 11 (38%) of the 29 athletes had moderate laryngeal obstruction and received advice on breathing technique; among them, 8 (73%) reported exercise-induced dyspnea during the last 12 months. At follow-up, 8 (73%) of the athletes receiving advice on breathing technique had an unchanged supraglottic score. Three (17%) of the 18 athletes with no or mild laryngeal obstruction at baseline had moderate supraglottic obstruction at follow-up, and none of the 3 reported exercise-induced dyspnea.

**Conclusions:**

In athletes with repeated testing, CLE scores remain mostly stable over 3–24 months even with advice on breathing technique to those with EILO. However, there is some intraindividual variability in CLE scores over time.

**Trial registration:**

ISRCTN, ISRCTN60543467, 2020/08/23, retrospectively registered, ISRCTN – ISRCTN60543467: Investigating conditions causing breathlessness in athletes.

## Background

Exercise-induced laryngeal obstruction (EILO) is an important and possibly treatable cause of exertional dyspnea, affecting about 5.7%–7.6% of a general population and 8.1%–27% of athletic populations [1–4]. EILO causes respiratory symptoms of especially inspiratory character during high-intensity effort [5, 6]. It is important to test for EILO in patients with exercise-induced dyspnea, because EILO is often mistaken for asthma and the two conditions often co-exist [1, 2, 7]. Because symptoms of EILO abate quickly upon exercise cessation, diagnosis requires visualizing the larynx during, or in close association to, a maximal bout of exercise [5, 8, 9]. A common test used in the clinical and research setting is the continuous laryngoscopy during exercise (CLE) test [10]. The test involves the use of a flexible nasolaryngoscope during a maximal effort exercise bout, usually on a treadmill or bicycle [11, 12]. The laryngeal obstruction at the glottic (i.e., vocal cord) and supraglottic levels is then graded, usually by an observer using a 0–3-point visual grade score [12, 13].

Only one study has described the natural course of EILO diagnosed by CLE in teenagers given advice on breathing techniques and having predominantly supraglottic EILO. CLE-scores were normalized in 3 out 14 subjects, respiratory symptom severity decreased, and the teenagers reported a higher ability to perform exercise over a period of 2–5 years [14]. Non-surgical treatment of EILO may be most effective in glottic EILO, as indicated in two recent small studies focusing on inspiratory muscle training and on breathing techniques combined with cognitive behavioral therapy [15, 16]. Nonetheless, advice on breathing techniques during exercise is recommended to patients with mild EILO regardless of the location of obstruction, whereas surgical treatment is an option in selected highly motivated patients with severe supraglottic EILO [9, 14, 17, 18]. Other interventions such as biofeedback, speech therapy, and laryngeal control therapy may also have an effect on EILO [8, 9, 19–21]. However, the few longitudinal studies on EILO patients have not included asymptomatic controls. Indeed, there is currently a lack of evidence regarding treatment of EILO, and no randomized controlled trials of treatment have been published.

A recent review on EILO in highlighted our scarce knowledge on the natural history and prognosis of EILO [22].

We need more understanding of the validity of the CLE test, the natural history of EILO and effects of treatment. The aim of this prospective study was to examine the variability of CLE scores in athletes, irrespective of EILO diagnosis, treatment, and respiratory symptoms.

## Materials and methods

### Study design and study population

This prospective screening study on EILO diagnosed by CLE test with one baseline visit and one follow-up was open to elite cross-country skiers (competing in cross-country skiing, biathlon, or ski-orienteering) aged 15–35 years. Elite skiers were defined as skiers belonging to Swedish national teams, national elite upper secondary sport schools, universities with elite sports agreement, or had competed at national or international level during the last 12 months. Information about the study was distributed to coaches and medical personnel of the Swedish Ski Association, Swedish Biathlon Federation, and Swedish National Elite Sports Schools in the county of Jämtland Härjedalen. Information about the study was also distributed via advertisement at suitable facilities.

A total of 98 athletes (including 89 cross-country skiers or biathletes) participated in the baseline visit and were screened for EILO, allergy, and asthma [23]. The aim of the follow-up was to re-examine these 98 athletes after 6 months irrespective of airway symptoms and diagnoses. Twenty-nine of the 98 participated in the follow-up. Due to the competition calendar and logistics, the mean duration between first visit and follow-up was 8.4 (min 3, max 23) months.

### Setting

The study was carried out at Östersund Hospital, Sweden, in 2015–2017. Both visits included a medical history, clinical examination, dynamic spirometry, eucapnic voluntary hyperventilation (EVH) test, CLE test, and questionnaires.

### Study procedures

#### Spirometry and EVH test

Dynamic spirometry was performed using a Spirare 3 (Diagnostics, Norway) or Jaeger Vyntus IOS (CareFusion, Germany) device [24]. EVH testing was conducted using the AILOS Asthma Test (Ailos Medical AB, Sweden) or Eucapsys (SMTEC, Switzerland). Participants were instructed to hyperventilate dry air with 5% CO_2_ (Carboair, Air Liquid GAS, Sweden) for 6 min with a target minute ventilation set at 85% of maximum voluntary hyperventilation (30 * FEV_1_ [Forced Expiratory Volume] in 1 s) [25]. FEV_1_ was measured before and at 3, 5, 10, 15, and 20 min post EVH, with the highest FEV_1_ of two approved maneuvers recorded. Exercise-induced bronchoconstriction was defined as a ≥ 10% decrease in FEV_1_ after EVH [26]. Participants were instructed to avoid vigorous exercise, heavy meals, caffeine, and nicotine for 8 h before the EVH test. They also were instructed to refrain from using asthma medication before the test, within the following time frames: short-acting β2-agonists for 8 h, inhaled corticosteroids for 12 h, long-acting β2-agonists for 24 h, and leukotriene receptor agonists for 3 days. Participants had to be free from respiratory infection for at least 6 weeks before the test.

#### Questionnaire

The questionnaire was based on the European Community Respiratory Health Survey II (ECHRS II) and covered training background, medications, airway symptoms, allergies, asthma, and family history [27]. Key variables were defined as:

*Physician-diagnosed asthma:* “Yes” to “Have you ever had asthma?” and “Was it diagnosed by a medical doctor?”.

*Asthma medication last 12 months*: “Yes” to “Have you used any asthma medication (including inhalers, aerosols, or tablets) in the last 12 months?

*Current asthma*: Having physician-diagnosed asthma and use of asthma medication in the last 12 months.

*Exercise-induced dyspnea* (EID): “Yes” to the question “Have you had any shortness of breath following exercise, during the last 12 months?”.

#### Continuous laryngoscopy during exercise test

The CLE test was performed according to Heimdal et. Al [10]. The larynx was continuously visualized with a nasal fiber-optic nasolaryngoscope (FNL –7RP3, Pentax, Tokyo, Japan) and video-recorded during exercise on a treadmill (Intelligent Suspension 3, Cybex, US). Naphazoline-lidocaine was used for local anesthesia and dilatation of the nasal cavity pre insertion of the nasolaryngoscope. Speed or inclination of the treadmill was increased at every minute after a 3-min warm-up, to reach maximal effort at 6–9 min of incremental exercise. Speed increased by 1 km/h up to a maximum of 15 km/h, and inclination by 2% up to a maximum of 8%. The intention was to exercise the athletes to exhaustion, but we set the lowest acceptable target to > 90% of estimated maximal heart rate. Heart rate was monitored using a chest strap (Polar, Finland).

Laryngeal obstruction at the glottic and supraglottic levels was assessed at 1 min after warm-up and at maximal effort, using an ordinal visual grade score (0–3 points; none (0), mild (1), moderate (2), severe (3)) according to Maat et. Al [13, 28]. EILO was defined as a CLE score of ≥ 2 points at the glottic or supraglottic level at maximal effort. CLE tests were performed and EILO was diagnosed at the discretion of the study physician (M.R) consultant in otorhinolaryngology, and a physiotherapist (C.B.). M.R. graded the laryngeal obstruction, blinded to results from other parts of the study and to whether the participant had reported any airway symptoms. A second blinded grading of the video laryngoscopies, without access to sound or external video recording, was done by resident physician in pediatrics T.I. A final CLE score was set by M.R. in case of disagreement.

*Respiratory distress* during the CLE test was graded by the observer ordinally as: “none”, “mild, with audible respiration”, “moderate, with stridor”, and “collapse or panic attack”. Any disagreement between the observers was solved by consensus. Except for the very beginning of the study, participants rated their *perceived physical exertion* according to the Borg rating of perceived exertion scale (RPE Scale), ranging from 6 (no exertion at all) to 20 (maximal exertion), immediately after cessation of the exercise [29].

#### Clinical treatment of study participants

All athletes participating in the study were cared for in the same manner as practiced in the clinical setting, and any recommendations were at the discretion of the study physicians. For participants with significant laryngeal obstruction, this included a review of laryngeal findings on video and self-care advice on breathing techniques to use during exercise. For instance, they were advised to focus on diaphragmatic breathing through closed-lip-breathing and to modify the posture e.g. keeping the head high and lifted, and the shoulders low and retracted [30]. There was no follow-up between visits, such as a check on adherence to recommendations. No participant with EILO received speech therapy or surgery.

### Statistics

Data analyses were conducted using R Statistical Software (version 3.6.1). The chi-square test was used for categorical variables. The Wilcoxon signed-rank test was used to compare non-parametric data at baseline versus follow-up. Results were considered statistically significant at a *P*-value of < 0.05.

## Results

### Population characteristics

Of the 29 athletes (15 females) who completed baseline and follow-up visits, 27 were skiers (cross-country skiers or biathletes), one was an elite orienteer, and one was an elite track-and-field athlete. Prior to the baseline visit, none of the athletes had been diagnosed with EILO, and none had ever smoked regularly. Baseline characteristics are presented in Table 1. The 29 athletes did not differ from the 69 that did not participate in the follow-up regarding age or the proportion of females, physician-diagnosed asthma, current asthma, EID, or EILO (≥ 2 points at the supraglottic or glottic level at maximal effort) (Table 2).

**Table 1:** Study population, *n* = 29, at baseline

Age, years, mean (min–max)	19 (16–27)
FEV_1_% of predicted, mean (SD)	104 (12.5)
FVC % of predicted, mean (SD)	100 (11.0)
BMI, mean (SD)	21.8 (1.7)

*FEV*_*1*_ forced expiratory volume in one second, *FVC* forced vital capacity, *BMI* body mass index

**Table 2:** A comparison between the athletes that participated in the follow-up and those that did not

	**Follow-up, *n* = 29**	**No follow-up, *n* = 69**	**All, *n* = 98**	***P*-value**
Males	14 (48%)	33 (47%)	47 (48%)	0.918
Age, median (quartile 1-quartile 3)	18 (17–19)	18 (17–21)	17 (18–20)	0.568
EID	11 (38%)	20 (29%)	30 (31%)	0.361
Physician-diagnosed asthma	12 (41%)	28 (40%)	40 (41%)	0.899
Current use of asthma medication	10 (34%)	22 (31%)	32 (33%)	0.767
EILO	11 (38%)	16 (23%)	26 (28%)	0.170
**Supraglottic**	11 (38%)	16 (23%)	27 (28%)	
**Glottic**	0 (0)	0 (0)	0 (0)	
**Borg RPE**	16 (16–16.5)	17 (16–17)	16.75 (16–17)	

*EID* exercise-induced dyspnea at baseline

*EILO* ≥ 2 points at the supraglottic or glottic level at maximal effort at baseline

*Borg RPE* Borg RPE during baseline CLE, median (quartile 1-quartile 3)

### Baseline

Of the 29 athletes, 11 (38%) athletes (ten females) had moderate supraglottic obstruction, visual grade score of 2, at maximal effort during the baseline CLE test (Table 3). These were given self-care advice on breathing techniques. Three (27%) out of the 11 athletes with moderate obstruction did not report any exercise-induced dyspnea in the previous 12 months. The 18 athletes without EILO at baseline are described in Table 4. None of the 29 athletes had glottic EILO, but two had mild glottic obstruction.

**Table 3:** The eleven athletes with laryngeal obstruction at baseline receiving advice on breathing techniques and their status at follow-up

	**Baseline**								**Follow-up**					
Sex	Age	Current asthma	FEV_1_ post EVH^a^	CLE score glottic + supraglottic	EID	Peak heart rate during CLE test	Borg RPE during CLE test	Months between visits	Current asthma	FEV_1_ post EVH^a^	CLE score glottic + supraglottic	EID	Peak heart rate during CLE test	Borg RPE during CLE test
**Female**	16	No	5.4	1 + 2	No	187	NA	5	No	-2.0	0 + 2	Yes	190	15
**Female**	18	No	4.2	0 + 2	No	189	NA	9	No	-12.1	0 + 2	Yes	187	15
**Female**	16	No	-7.7	0 + 2	Yes	186	NA	6	No	-1.8	0 + 2	No	174	14
**Female**	**17**	Yes	-5.7	0 + 2	Yes	194	16	5	Yes	-10.3	0 + 1	Yes	205	16
**Male**	19	Yes	0.3	0 + 2	Yes	190	16	12	No	-1.9	0 + 2	No	192	17
**Female**	26	Yes	-3.6	0 + 2	Yes	182	17	3	Yes	-6.1	0 + 2	Yes	178	18
**Female**	16	No	-7.8	0 + 2	Yes	192	16	11	No	-0.6	0 + 2	No	190	16
**Female**	16	No	-13.8	0 + 2	No	200	NA	23	No	-8.1	0 + 2	No	184	16
**Female**	**18**	No	-2.0	0 + 2	Yes	205	NA	6	No	-3.5	0 + 1	Yes	205	15
**Female**	**20**	Yes	-4.0	0 + 2	Yes	176	NA	11	Yes	0.5	0 + 1	Yes	178	16.5
**Female**	20	Yes	-9.3	0 + 2	Yes	187	16.5	11	Yes	-9.3	0 + 2	Yes	192	17

^a^Maximal % change compared with baseline. Current asthma = physician-diagnosed asthma and asthma medication use in the last 12 months

*FEV*_*1*_ forced expiratory volume during the first second, *EVH* eucapnic voluntary hyperventilation test, *CLE* continuous laryngoscopy during exercise test; glottic and supraglottic scores at maximal effort, *EID* exercise-induced dyspnea

**Table 4:** Athletes with no-to-mild laryngeal obstruction at baseline, and their results at the follow-up visit

	**Baseline**						**Follow-up**			
Sex	Age	Current asthma	FEV_1_ post EVH^a^	CLE score glottic + supraglottic	EID	Months between visits	Current asthma	FEV_1_ post EVH^a^	CLE score glottic + supraglottic	EID
**Male**	17	Yes	-2.7	1 + 1	No	3	Yes	-2.6	1 + 1	No
**Female**	16	No	-8.3	0 + 1	No	9	No	-8.4	0 + 1	No
**Female**	18	No	-3.4	0 + 1	No	*9*	No	-3.7	0 + 1	No
**Male**	**17**	No	-3.6	0 + 1	No	4	No	0.0	0 + 2	No
**Female**	23	Yes	-6.0	0 + 1	No	10	Yes	-5.9	0 + 0	No
**Female**	**19**	No	-3.4	0 + 1	No	8	No	-2.4	0 + 2	No
**Female**	24	No	-7.0	0 + 1	Yes	11	No	-5.3	0 + 1	No
**Male**	19	No	-3.4	0 + 1	No	6	No	-3.4	0 + 1	No
**Male**	19	Yes	-12.7	0 + 1	No	6	Yes	-2.2	0 + 1	Yes
**Male**	16	No	-9.2	0 + 1	Yes	6	No	< -10.0^b^	0 + 1	No
**Male**	17	Yes	-1.6	0 + 1	No	8	Yes	-2.9	0 + 1	No
**Male**	**27**	No	-5.6	0 + 1	No	21	No	-12.1	0 + 2	No
**Male**	18	No	-13.0	0 + 1	No	6	Yes	-10.5	0 + 0	No
**Male**	16	No	-3.1	0 + 0	No	8	No	-7.0	0 + 1	No
**Male**	19	No	-4.4	0 + 0	No	5	No	< -10.0^b^	0 + 0	No
**Male**	18	Yes	-3.4	0 + 0	Yes	3	Yes	< -10.0^b^	0 + 0	Yes
**Male**	18	No	-9.5	0 + 0	No	7	No	-6.5	0 + 1	No
**Male**	20	No	-3.3	0 + 0	No	13	No	1.4	0 + 0	No

*FEV*_*1*_ forced expiratory volume during one second, *EVH* eucapnic voluntary hyperventilation test, *CLE* continuous laryngoscopy during exercise test; glottic and supraglottic scores include scores only as maximal effort, *EID* exercise-induced dyspnea

^a^Maximal % change compared to baseline

^b^ Absolute values missing due to equipment failure, EVH tests negative according to case report files. Current asthma = physician-diagnosed asthma and asthma medication use in the last 12 months

Exercise-induced dyspnea was more common in the athletes with moderate obstruction compared to those without (73% vs. 17%, *P* = 0.003). Only three of 29 athletes had observer-rated respiratory distress during the baseline CLE test. Their respiratory distress was rated as “*mild, with audible respiration*” and all three had EILO. No athlete had respiratory distress of “*moderate, with stridor*” or “*collapse or panic attack*” character.

At the baseline CLE test, median (interquartile range [IQR]) peak heart rate was 96 (94–98) % of estimated maximum heart rate.

### Follow-up

Training hours/week at follow-up did not differ from those at baseline (median (quartile 1-quartile 3)), the athletes trained median (quartile 1-quartile 3) 12 (10–14) vs 12 (10–13) hours/week, *P* = 0.130. Training hours for the athletes with EILO at baseline was also similar between follow-up and baseline, 10 (10–12) vs 10 (10–12), *P* = 1, In the 11 athletes with moderate obstruction at first visit, 8 (73%) had the same supraglottic score at follow-up, and three athletes were graded with mild obstruction (Table 3). Seven out of 11 athletes with moderate obstruction at follow-up did not report any EID in the previous 12 months. In the 18 athletes with none or mild obstruction at first visit, three athletes had moderate obstruction at follow-up (Table 4).

Two of the three athletes with observer-graded respiratory distress persisted as “*mild, with audible respiration*” at the second CLE test. No other athlete was observed with respiratory distress at follow-up.

At follow-up, median (IQR) peak heart rate was 94 (93–97) % of estimated maximum heart rate, which did not significantly differ (*P* = 0.112) from peak heart rate at baseline.

### Asthma

Among the 10 athletes with current asthma at baseline, one had EIB according to the EVH test, and six reported having had an asthma attack during the last 12 months. At follow-up, two out of nine athletes with current asthma had EIB. In total, six (21%) athletes had EIB at baseline or follow-up. Three out of five athletes with both current asthma and EILO at baseline had stopped using asthma medication at follow-up. These five athletes had negative EVH tests at both visits.

## Discussion

### Main summary

This prospective study assessed the variability in CLE scores over time in athletes irrespective of grade of laryngeal obstruction, respiratory symptoms, and intervention. We found that the CLE scores mainly remained stable over time. However, 27% of those with moderate supraglottic obstruction at baseline and given advice on breathing techniques had mild obstruction at follow-up, and 17% of those with no-to-mild supraglottic obstruction at baseline had moderate obstruction at follow-up. None of the athletes had significant glottic obstruction at baseline or follow-up.

### CLE scores remain mostly stable in athletes treated for EILO

In the present study, participants with moderate supraglottic obstruction were given advice on breathing techniques to use during exercise, at the discretion of the study physician. The change in laryngeal obstruction between visits in those with moderate obstruction in the present study is similar to that in 14 teenage patients given advice on breathing techniques for supraglottic EILO, among whom 21% (*n* = 3) had normalized laryngeal function after 2–5 years [14]. Two recent studies using CLE scores to assess treatment outcome found that inspiratory muscle training and physiotherapy may have an effect, especially on glottic EILO [15, 16]. However, many studies employ methods other than the CLE test to evaluate laryngeal obstruction, often only assessing the glottic level of obstruction post exercise, making comparisons with the present findings difficult [8, 31]. Also, while subjective improvement is important, more objective data related to laryngeal obstruction is needed to give us a better understanding of EILO. To what extent different factors contribute to the remission or incidence of EILO is unknown, and this underlines the need for randomized controlled trials of treatments in these populations.

The breathing technique intervention in the present study appear to have had a limited effect on laryngeal obstruction and respiratory symptoms. The effectiveness of a breathing technique intervention on laryngeal obstruction is largely unknown due to the lack of randomized controlled trials. It is therefore difficult to know whether the apparent lack of effect was a result of poor adherence, an ineffective intervention, or participation bias. Nevertheless, our results indicate that supraglottic EILO in elite skiers does not resolve to any large extent, neither spontaneously nor upon advice on breathing, when reassessed within 2 years.

### EILO and sex

Of the 11 athletes with EILO at follow-up, 10 were females. Several studies have shown that EILO is more common in females than men [3, 7, 8, 32]. Other studies do not find sex differences [1, 2]. One explanation for the female dominance may be due to differences in laryngeal size. Prepubertal laryngeal size is similar in boys and girls, but pubertal growth differences result in a larger male larynx [33, 34]. Others have shown that adolescent female athletes with EILO have a reduced respiratory resistance, perhaps due to reduced laryngeal tone [35, 36]. A simpler explanation to the sex difference in the present study is participation bias. We cannot rule out the possibility that female athletes with persistent EILO were more interested, due to unknown reasons, to participate in the follow-up CLE test than the male athletes with EILO.

### Screening of EILO may detect asymptomatic supraglottic obstruction

A strength and novelty of the present study is the prospective follow-up of athletes without EID or EILO. In most (83%) of the subjects with no-to-mild laryngeal obstruction, the baseline CLE scores persisted at follow-up. However, three of these athletes had incident moderate supraglottic obstruction at follow-up. Interestingly, all three did not have EID in the previous 12 months and did not have respiratory distress as graded by the observer during the CLE test. Also, three out of 8 athletes with EILO and EID at baseline, reported no EID at follow-up despite persistent laryngeal obstruction fulfilling the study criteria for EILO.

Many epidemiological studies have defined EILO purely based on laryngeal obstruction during a CLE test and many with EILO did not report any self-reported exercise-related respiratory symptoms [2, 3]. Screening has also shown that a substantial proportion of athletes with EILO do not report exercise-induced symptoms [1]. So, asymptomatic subjects with EILO are not uncommon.

Furthermore, we cannot be certain that athletes reporting *shortness of breath following exercise during the last 12 months*, had these symptoms purely due to EILO. Although breathlessness is a typical symptom of EILO, EILO may also induce other symptoms such as chest tightness and throat discomfort not covered in our questionnaire [3, 5]. Thus, we may have falsely described some athletes with EILO as asymptomatic. Self-reported respiratory symptoms are poor at predicting EILO and also of limited value in discerning EILO from asthma [1–3, 7, 37]. However, subjects with EILO have increased work of breathing [38]. Thus, it remains to be established whether mild laryngeal obstruction in an asymptomatic subject constitutes a pathological or normal laryngeal response to high-intensity exercise.

### Asthma and EIB

Very few (one out of ten) athletes with current asthma had EIB, indicating a possible overdiagnosis of asthma in the study population [39]. Cross-country skiers are known to have a high prevalence of asthma, probably because of prolonged inhalation of cold dry air leading to airway hyperreactivity [40, 41]. However, the high prevalence of EILO in our study population, indicates that objective testing for both asthma and EILO is important in athletes with exercise-induced symptoms. Also in Norway many athletes diagnosed with EILO use asthma medication and many without objective evidence of variable broncho-obstruction according to retrospective reviews [42].

## Limitations

The CLE test protocol used may have led to false low visual-grade scores. Firstly, the target heart rate during the CLE test of 90% of the estimated maximum may simply have been too low to induce EILO in the present study population including athletes competing at the highest national or international level [22]. If the participants had exerted even harder we might have detected more glottic EILO, which tends to develop after supraglottic obstruction [13]. Secondly, the visual grade score might underestimate glottic obstruction [43].

There was a risk of participation bias at both baseline and follow-up. Our study had a high proportion of cross-country skiers with EID at baseline. Recruitment via open invitation possibly attracted athletes with respiratory symptoms, inducing a risk of overestimating the prevalence of EILO and asthma [39]. Furthermore, among the athletes participating in the follow-up, a higher proportion had EID compared with the whole population of cross-country skiers at baseline, indicating that athletes with respiratory symptoms may have been more prone to participate in the follow-up. This could mean that the present results are skewed towards athletes with respiratory symptoms, irrespective of laryngeal obstruction, and underestimate the effects of breathing advice for EILO.

The CLE scoring system has received criticism for not being a robust method of assessing laryngeal obstruction, which if true could have affected our results. Studies assessing its validity and reliability have come to different conclusions [13, 44]. The CLE test score has been found to be comparable to EILOMEA, a more objective method of assessing laryngeal obstruction that uses software to estimate the grade of obstruction from a still frame of the larynx taken during a CLE test [45]. Taken together, there is a need for objective methods with which to assess laryngeal obstruction for use during the CLE test.

One study has shown that athletes in subsequent CLE tests could exercise for a longer time before exhaustion [44]. Our results do not indicate any learning effect. The athletes’ CLE test performance, in terms of self-perceived exertion and peak heart rate percent of estimated maximum heart rate, was similar at baseline and follow-up.

## Conclusion

In athletes performing repeated diagnostic tests for EILO, the CLE test score is mostly stable over time, but there is some intra-individual variability in scores. In the present study, the incident cases of moderate supraglottic laryngeal obstruction did not have exercise-related respiratory symptoms, indicating a need to establish what constitutes a normal laryngeal response to exercise in athletes. This study also highlights the need for randomized controlled trials that objectively assess factors associated with treatment effects in patients with EILO.

## Data Availability

The datasets used and/or analysed during the current study are available from the corresponding author on reasonable request.

## Abbreviations

BMI: Body mass index
CLE: Continuous laryngoscopy during exercise
EID: Exercise-induced dyspnea
EILO: Exercise-induced laryngeal obstruction
EVH: Eucapnic voluntary hyperventilation
FEV_1_: Forced expiratory volume first second
FVC: Forced vital capacity
IQR: Interquartile range
RPE: Rating of perceived exertion
